# DO FUNCTIONAL ABILITY MEASURES IMPROVE THE ACCURACY OF A 12-MONTH MORTALITY PREDICTION MODEL?

**DOI:** 10.1093/geroni/igae098.2245

**Published:** 2024-12-31

**Authors:** Elizabeth Luth, Kathryn Bowles, Carlin Brickner, Oude Gao, Harivony Rakotoarivelo

**Affiliations:** Rutgers University, New Brunswick, New Jersey, United States; University of Pennsylvania, Philadelphia, Pennsylvania, United States; VNS Health, New York, New York, United States; VNS Health, New York, New York, United States; VNS Health, New York, New York, United States

## Abstract

Mortality prediction algorithms using administrative claims data can help identify seriously ill individuals eligible for palliative care. However, predefined measures of functional ability, which clinicians report as important for eligibility determinations, may be inadvertently omitted or underweighted in the algorithm general design. In response to clinician input, we developed measures of functional ability using claims data and examined their association with death: activities of daily living (ADL) support, feeding support, respiratory support, physical and occupational therapy (PT/OT), and skilled nursing. Descriptive statistics were calculated. Multivariable logistic regression tested associations between five functional ability measures, symptom management, and death for Medicare Advantage plan members on census during the July 2022-June 2023 period. Of the 6625 Medicare Advantage plan members, 32% identified as Black, 32% White, 9% as Asian, and 26% as Other races; 37% as Hispanic/Latino; 69% female; average age of 73.5 years (SD: 11.8). Of these, 186 (4.6%) died the following year. Having PT/OT was associated with 24% lower odds of death (aOR: 0.76, 95%CI: 0.62-0.85, p< 0001). Receiving respiratory support was associated with 45% higher odds of death (aOR: 1.45, 95%CI: 1.17-1.78, p< 0.001). ADL support, feeding support, and skilled nursing were not associated with death. These functional ability measures, while not directly measured in claims data, were successfully approximated. Oxygen and ventilation use and PT/OT were associated with 12-month mortality, in opposite directions. Future work should examine how other factors used by clinicians for PC eligibility determinations can be measured using administrative claims data and their associations with mortality.

